# Trametinib in combination with hydroxychloroquine or palbociclib in advanced metastatic pancreatic cancer: data from a retrospective, multicentric cohort (AIO AIO-TF/PAK-0123)

**DOI:** 10.1007/s00432-024-05954-5

**Published:** 2024-10-01

**Authors:** David Witte, Ina Pretzell, Timm M Reissig, Alexander Stein, Janna-Lisa Velthaus, Annabel Alig, Hanibal Bohnenberger, Maren Knödler, Annika Kurreck, Sabrina Sulzer, Georg Beyer, Klara Dorman, Tabea Fröhlich, Stefanie Hegenberg, Celine Lugnier, Anna Saborowski, Arndt Vogel, Sebastian Lange, Maximilian Reichert, Franziska Flade, Lioba Klaas, Kirsten Utpatel, Heiko Becker, Annalen Bleckmann, Klaus Wethmar, Anke Reinacher-Schick, Christoph Benedikt Westphalen

**Affiliations:** 1 Department of Hematology, Oncology and Palliative Care, St. Josef Hospital, Ruhr University Bochum, Bochum, Germany; 2 West German Cancer Center, University Hospital Essen, Essen, Germany; 3 Department of Medical Oncology, West German Cancer Center, University Hospital Essen, Essen, Germany; 4 Bridge Institute of Experimental Tumor Therapy, West German Cancer Center, University Hospital Essen, Essen, Germany; 5 Hematology-Oncology Practice Eppendorf, University Cancer Center Hamburg, Hamburg, Germany; 6 Department of Oncology, Hematology and BMT with Section Pneumology, University of Hamburg, Hamburg, Germany; 7 Department of Hematology, Oncology and Tumorimmunology, Charité-Universitätsmedizin Berlin, Freie Universität Berlin, Humboldt-Universität zu Berlin, Berlin, Germany; 8 Institute of Pathology, University Medical Center Göttingen, Göttingen, Germany; 9 Charité Comprehensive Cancer Center, Charité - Universitätsmedizin Berlin, Corporate Member of Freie Universität Berlin and Humboldt-Universität zu Berlin, Berlin, Germany; 10 Department of Gastroenterology, Gastrointestinal Oncology and Endocrinology, University Medical Center, Goettingen, Germany; 11 Medical Department II, LMU University Hospital, LMU Munich, Munich, Germany; 12 Bavarian Cancer Research Center (BZKF), Munich, Germany; 13 Department of Medicine III, University Hospital, LMU Munich, Munich, Germany; 14 German Cancer Consortium (DKTK), Partner Site Munich, Munich, Germany; 15 Department of Hematology, Hemostasis, Oncology and Stem Cell Transplantation, Hannover Medical School, Hannover, Germany; 16 Department of Gastroenterology, Hepatology, Infectious Diseases and Endocrinology, Hannover Medical School, Hannover, Germany; 17 TUM School of Medicine and Health, Department of Clinical Medicine, Clinical Department for Internal Medicine II, University Medical Center, Technical University of Munich, Munich, Germany; 18 Hematology Practice Probstheida, Strümpellstraße 42, Leipzig, Germany; 19 Department of Internal Medicine II, Faculty of Medicine, University Medical Center Freiburg, Faculty of Medicine, University of Freiburg, Freiburg, Germany; 20 Institute of Pathology, University Regensburg, Regensburg, Germany; 21 Department of Hematology, Oncology and Stem Cell Transplantation, Center for Personalized Medicine, Faculty of Medicine, University Medical Center Freiburg, University of Freiburg, Freiburg, Germany; 22 Department of Medicine A, Hematology, Oncology, Hemostaseology and Pneumology, University Hospital Münster, Münster, Germany

**Keywords:** Pancreatic cancer, MEK inhibitor, CDK inhibitor, Autophagy, Targeted therapy, Molecular guided treatment

## Abstract

**Background:**

Preclinical models of pancreatic cancer (PDAC) suggest a synergistic role for combined MEK and autophagy signaling inhibition, as well as MEK and CDK4/6 pathway targeting. Several case reports implicate clinical activity of the combination of either trametinib and hydroxychloroquine (HCQ) in patients with KRAS-mutant PDAC or trametinib with CDK4/6 inhibitors in patients with KRAS and CDKN2A/B alterations. However, prospective data from clinical trials is lacking. Here, we aim to provide clinical evidence regarding the use of these experimental regimens in the setting of dedicated precision oncology programs.

**Methods:**

In this retrospective case series, PDAC patients who received either trametinib/HCQ (THCQ) or trametinib/palbociclib (TP) were retrospectively identified across 11 participating cancer centers in Germany.

**Results:**

Overall, 34 patients were identified. 19 patients received THCQ, and 15 received TP, respectively. In patients treated with THCQ, the median duration of treatment was 46 days, median progression-free survival (PFS) was 52 days and median overall survival (OS) was 68 days. In the THCQ subgroup, all patients evaluable for response (13/19) had progressive disease (PD) within 100 days. In the TP subgroup, the median duration of treatment was 60 days, median PFS was 56 days and median OS was 195 days. In the TP subgroup, 9/15 patients were evaluable for response, of which 1/9 showed a partial response (PR) while 8/9 had PD. One patient achieved a clinical benefit despite progression under TP.

**Conclusion:**

THCQ and TP are not effective in patients with advanced PDAC harboring KRAS mutations or alterations in MAPK/CDKN2A/B.

## Introduction

Pancreatic ductal adenocarcinoma (PDAC) conveys a dismal prognosis and is expected to become the second most common cause of cancer-related mortality in the US and also in Germany by 2030 (Park et al. 2021; Quante et al. 2016). While molecularly guided treatment options have become standard of care in various malignancies, this has not been the case for PDAC, where cytotoxic chemotherapy remains the mainstay in the treatment of metastatic pancreatic cancer (Park et al. 2021). Based on registry data, common treatment regimens in Germany are FOLFIRINOX (5-FU/folinic acid/irinotecan/oxaliplatin), Gemcitabin/nab-Paclitaxel, nanoliposomal irinotecan/5-FU/folinic acid as well as oxaliplatin/5-FU/folinic acid (OFF) (Marschner et al. 2024; Hegewisch-Becker et al. 2022). These combinations are recommended by the German national guideline for PDAC, however the guideline does not recommend a specific sequence of these treatment regimens (German guideline for exocrine pancreatic cancer version 3.0, 2024). In selected subgroups, some progress regarding targeted therapy has been made. Olaparib maintenance can be applied after response to platinum-based therapy in patients with metastatic PDAC harboring a germline BRCA1/2 mutation (Golan et al. 2019). The most common driver mutations in the development of PDAC tumorigenesis are activating mutations in the KRAS proto-oncogene with a rate of about 90%, as well as inactivating mutations in the tumor suppressor genes TP53, SMAD4 and CDKN2A (Grant et al. 2016). These core mutational drivers of PDAC have largely remained elusive to targeted treatment approaches. However, in the small subgroup of PDAC lacking a KRAS mutation, other driver mutations have recently been identified, with many of these being susceptible to targeted treatment approaches, like NTRK (Doebele et al. 2020; Hong et al. 2020), NRG1 (Schram et al. 2022), RET(Subbiah et al. 2022) and mismatch repair deficiency (dMMR) (Coston et al. 2023). While targeting KRAS has been a challenging task, the recent emergence of mutation-specific KRAS-G12C-directed inhibitors sotorasib and adagrasib has opened a promising perspective on targeted treatment of PDAC (Strickler et al. 2023; Bekaii-Saab et al. 2023), yet due to the low prevalence of KRAS-G12C mutations in PDAC, these approaches so far have limited impact on the treatment of the majority of PDAC patients (Khan et al. 2023). However, novel compounds targeting other KRAS isoforms as well as Pan-KRAS and Pan-RAS inhibitors are on the brink of later clinical development. Despite these recent advances, no approved targeted treatment options apart from olaparib and the rarely used gemcitabine/erlotinib regimen are available in Germany, and the German national guideline does not specifically recommend comprehensive molecular profiling of PDAC patients apart from gBRCA1/2 testing, resulting in a variety of center-specific approaches for comprehensive molecular profiling of PDAC patients (German guideline for exocrine pancreatic cancer version 3.0, 2024; Dorman et al. 2023). Within the centers for personalized medicine, Germany is aiming to build and harmonize a nation-wide precision oncology platform for patients with advanced cancers, which will hopefully optimize the access of patients to personalized treatment options and to molecularly guided clinical trials (Illert et al. 2023).

Recently, preclinical data, and case reports of two targeted treatment approaches in advanced PDAC have emerged: trametinib in combination with the autophagy inhibitor hydroxychloroquine (HCQ) in PDAC harboring KRAS mutations, as well as trametinib in combination with CDK4/6 inhibitors in PDAC with concomitant KRAS and CDKN2A/B alterations (Xavier et al. 2021; Kato et al. 2021). Trametinib is a selective, oral inhibitor of the mitogen-activated protein kinases MEK1 and MEK2 downstream of KRAS in the canonical RAS-RAF-MEK-ERK signaling pathway, thereby inhibiting proliferative signaling and cell cycle progression mediated by mutant KRAS (Gilmartin et al. 2011). Trametinib is approved for use in malignant melanoma, however it has failed to provide significant clinical benefit in metastatic pancreatic cancer in a phase II clinical trial in combination with gemcitabine (Infante et al. 2014). In preclinical models, autophagy has been found as a potential mode of resistance towards inhibition of MEK-ERK signaling. Autophagy pathways have been described to be upregulated in cell culture and xenograft models of pancreatic cancer in response to inhibition of MEK-ERK signaling downstream of mutant KRAS (Bryant et al. 2019). In preclinical models, trametinib combined with autophagy inhibitors chloroquine or hydroxychloroquine showed a synergistic anti-tumorigenic effect (Kinsey et al. 2019). Based on these results, several case reports of patients with advanced PDAC successfully treated with hydroxychloroquine (HCQ) in combination with trametinib were published (Kinsey et al. 2019; Xavier et al. 2021; Wu et al. 2021).

Another mechanism of resistance towards MEK-ERK inhibition in PDAC is the concomitant occurrence of other driver mutations of the cell cycle, like CDKN2A/B alterations. Inactivation of the tumor suppressor gene CDKN2A is among the most frequent alterations found in PDAC, often cooccurring with KRAS mutations (Grant et al. 2016). CDKN2A and the related gene CDKN2B encode the proteins p16 and p15 which inhibit progression of the cell cycle by binding to cyclin-dependent kinases CDK4 and CDK6, thereby inhibiting cell proliferation. A molecular rationale exists for targeting CDK4/6 in case of CDKN2A/B loss or inactivation, yet the CDK4/6 inhibitor palbociclib did not show a meaningful benefit in a cohort of patients with CDKN2A-altered pancreatic or biliary cancer (Al Baghdadi et al. 2019). Kato et al. found co-alterations of RAS and cell cycle genes in 31.1% in a NGS data set of 1,937 patients with diverse tumors, and co-alterations of RAS and cell cycle genes showed a significant correlation with worse overall survival (Kato et al. 2020). Targeting both the MEK-ERK pathway and CDK4/6 with the MEK inhibitor selumetinib combined with the CDK4/6 inhibitor palbociclib was shown to have a synergistic antitumoral effect in a lung cancer xenograft model with concomitant RAS and CDKN2A alterations (Zhou et al. 2017). The combination of the MEK inhibitor PD0325901 with palbociclib also showed a synergistic effect in KRAS- and BRAF-mutant colorectal cancer cells both in vitro and in a xenograft model (Pek et al. 2017). Based on this preclinical work, Kato et al. reported data from 9 patients with KRAS or BRAF mutations cooccurring with CDKN2A/B alterations, 6 of which were PDAC patients. Combined treatment with trametinib and palbociclib, together with dabrafenib in two non-PDAC cases, resulted in a clinical benefit rate of 56% (5/9 patients), with two PDAC patients achieving a partial response (PR, 2/9 patients) with a progression-free survival (PFS) of 9 and 17.5 + months, respectively (Kato et al. 2021).

Based on the data outlined above, several cancer centers in Germany have in the past recommended trametinib/HCQ (THCQ) and trametinib/palbociclib (TP) as experimental treatments for patients with advanced PDAC without other treatment options available. These treatment regimens were recommended by molecular tumor boards (MTB) within structured precision oncology programs, based on molecular profiling for KRAS and CDKN2A/B alterations. Here, we report a pooled case series to provide real-world evidence discouraging the use of these regimens in advanced PDAC.

## Methods

### Patients and treatment

This study was conducted as a retrospective, multicentric data analysis. Patients matching the inclusion criteria were retrospectively identified at 11 participating cancer centers in Germany by searching electronic medical records.

The inclusion criteria were as follows:

1) diagnosis of pancreatic ductal adenocarcinoma.

2) combined treatment with a MEK inhibitor and hydroxychloroquine or.

3) combined treatment with a MEK inhibitor and a CDK4/6 inhibitor.

Patients with a documented objection against data evaluation for research purposes were excluded. Data was anonymized at the local cancer center and then centrally pooled for analysis.

All patients were treated based on recommendations of a local MTB and received either THCQ or TP after cost coverage was granted by the respective insurance company. Treatment was performed at the discretion of the local physicians as per institutional guidelines.

### Data collection and analysis

Clinical characteristics were evaluated in a descriptive manner. All clinical and response data as well as toxicity data was collected by the local investigators from reviewing available electronic medical records, using an electronic case report form provided by the central investigator team. The total number of MTB recommendations for either THCQ or TP were also collected by review of local MTB protocols. Duration of treatment was defined as the time from first dose to last dose of treatment. Progression-free survival (PFS) was defined as the time from first dose of treatment to clinical or radiographic progression or death from any cause, whichever occurred earlier. Overall survival (OS) was defined as the time from first dose of treatment to death from any cause. PFS and OS were calculated by Kaplan-Meier analyses in IBM SPSS Statistics version 26. Radiographic response was evaluated by review of real-world radiographic records by the local investigators; as these radiographic records were mostly not prepared according to RECIST1.1 criteria, radiographic response was defined as follows: partial remission (PR), a partial but clinically meaningful shrinkage of tumor manifestations; stable disease (SD), no significant change in the size as well as no new tumor manifestations; and progressive disease (PD), clinically meaningful increase in size or occurrence of new tumor manifestations. Clinical benefit was defined as a relief of tumor-associated symptoms regardless of radiographic response.

### Ethics approval

This study was approved by the ethics committee of Ruhr-University Bochum (approval nr. 23-7809). At the time of data analysis, most patients were deceased, therefore obtaining informed consent was not possible; also, obtaining informed consent was not required by the ethics committee for this study due to the use of retrospective and anonymized data. Additionally, several of the patients in this study were also included in local registries and had previously consented to data use for research purposes.

## Results

### Patients and demographics

In total, we identified 34 patients who had been treated with either THCQ or TP between July 2019 and October 2023 across eleven cancer centers. 19 patients had been treated with THCQ, and 15 patients had been treated with TP. None of the patients had received other MEK or CDK inhibitors than trametinib or palbociclib.

The median age across the whole cohort was 62 years. 25 patients (73,5%) were male and 9 (26,5%) were female. 33 out of 34 patients suffered from metastatic disease upon initiation of trametinib/HCQ or trametinib/palbociclib. Patients in the trametinib/HCQ subgroup had received a median of 3 prior treatment lines, while patients in the trametinib/palbociclib subgroup had received a median of 2 prior treatment lines. The most common first-line treatment regimen was FOLFIRINOX (20/34 patients), while the most common second-line regimen was gemcitabine/nab-paclitaxel (22/34 patients). The median ECOG performance score upon initiation of trametinib/HCQ or trametinib/palbociclib was ECOG 1 (range 0–2; 5/34 n.a.). Demographics are summarized in Table 1.

**Table 1:** Patient characteristics and demographics

		All patients (*n* = 34)	Trametinib/HCQ (*n* = 19)	Trametinib/ Palbociclib (*n* = 15)
**Age**, **years**		62 (32–81)	61 (32–81)	63 (43–70)
**Sex**, **n (%)**				
	Male	25 (73.5%)	13 (68.4%)	12 (80%)
	Female	9 (26.5%	6 (31.6%)	3 (20%)
**Previous treatment lines**, **median (range)**		3 (1–7)	3 (2–7)	2 (1–6)
	1 previous treatment line, n (%)	2 (5.9%)	0 (0%)	2 (13.3%)
	2 previous treatment lines, n (%)	14 (41.2%)	5 (26.3%)	9 (60%)
	3 previous treatment lines, n (%)	8 (23.5%)	7 (36.8%)	1 (6.7%)
	4 previous treatment lines, n (%)	4 (11.8%)	3 (15.8%)	1 (6.7%)
	> 4 previous treatment lines, n (%)	6 (17,7%)	4 (21.1%)	2 (13.3%)
**Median ECOG PS**		1	1	1
	ECOG 0, n (%)	4 (11.8%	2 (10.5%)	2 (13.3%)
	ECOG 1, n (%)	21 (61.8%)	13 (68.4%)	8 (53.3%)
	ECOG 2, n (%)	4 (11.8%)	0 (0%)	4 (26.7%)
	n.a., n (%)	5 (14.7%	4 (21.1%)	1 (6.7%)
**UICC stage**				
	UICC IV, n (%)	33 (97.1%)	18 (94.7%)	15 (100%)
	n.a., n (%)	1 (2.9%)	1 (5.3%)	0 (0%)
**KRAS status**				
	KRAS mutated, n (%)	34 (100%)	19 (100%)	15 (100%)
**CDKN2A status**				
	CDKN2A alteration/loss, n (%)	16 (47.1%)	1 (5.3%)	15 (100%)
	CDKN2A wt, n (%)	10 (29.4%)	10 (52.6%)	0 (0%)
	n.a., n (%)	8 (23.5%)	8 (42.1%)	0 (0%)

All 34 patients harbored a KRAS mutation, the most frequent of which was KRAS-G12D (18/34); one patient had a KRAS-G12R mutation. In the TP subgroup, all 15 patients harbored concomitant CDKN2A alterations, while only one patient in the THCQ subgroup featured a known CDKN2A alteration; for 8/19 patients of THCQ subgroup, information on CDKN2A status was not available (Table 1).

We also evaluated the frequency of MTB recommendations of either THCQ or TP across the participating cancer centers. In total, 167 MTB recommendations for THCQ and TP were reported by 8 participating centers between 2020 and 2023, while only 34 of these recommendations resulted in actual treatment of patients.

### Treatment and outcome in the trametinib/HCQ (THCQ) cohort

Out of the 34 patients identified, 19 patients received treatment with THCQ. The most common starting dose of HCQ in this subgroup was 400 mg/d (11/19 patients). Doses of HCQ were increased in several patients, with 5 patients receiving a maximum dose of 800 mg/d and 5 patients receiving a maximum dose of 1200 mg/d. 16 out of 19 patients in the trametinib/HCQ subgroup received trametinib at a dose of 2 mg/d throughout their treatment. 12 out of 19 patients received trametinib/HCQ without interruption, while in 4 out of 19 patients an interruption of treatment was reported.

**Fig. 1:**
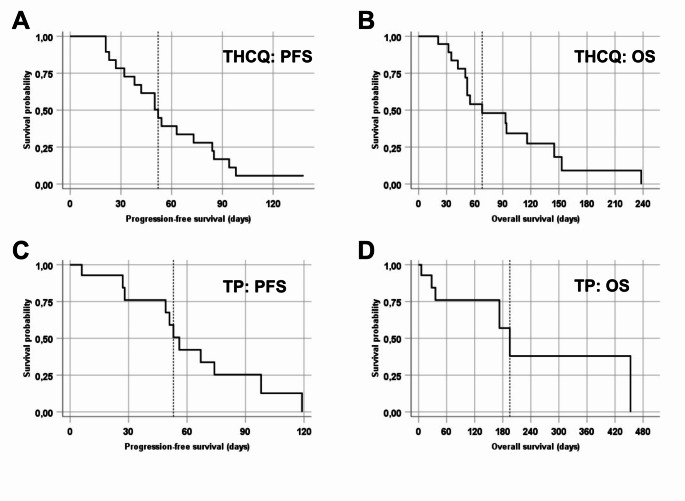
Kaplan-Meier plots of the survival of patients treated with trametinib/HCQ (THCQ) or trametinib/palbociclib (TP). **A**: Progression-free survival (PFS) of patients treated with THCQ; **B**: Overall survival (OS) of patients treated with THCQ; **C**: PFS of patients treated with TP; **D**: OS of patients treated with TP. The dotted vertical line indicates the respective median PFS and OS

The median duration of treatment in the trametinib/HCQ subgroup was 46 days, with the longest duration of trametinib/HCQ treatment being 86 days (range 9–86 days; 3/19 patients: n.a.). The median PFS in the trametinib/HCQ subgroup was 52 days (range 19–98 days; 95% CI: 38.3–65.7; 2/19 patients: n.a.) (Fig. 1A). The median OS in the trametinib/HCQ subgroup was 68 days (range 21–238 days; 95% CI: 16.4–119.6; 4/19 patients: n.a.) (Fig. 1B). For all 13 out of 19 patients in the trametinib/HCQ subgroup who were evaluable for response, progressive disease (PD) was reported, while for the remaining 6 out of 19 patients no data on best response was available; however, no clinical benefit of any kind was reported for the patients in the trametinib/HCQ subgroup. Notably, the patient with a KRAS-G12R mutation had PD after 23 days and died 35 days after initiation of treatment. The six patients for whom no response data was available did not receive follow-up staging for different reasons. Of these six patients, one patient died 30 days after initiation of treatment; in two patients, treatment was discontinued due to patient request; and in three patients, treatment was discontinued due to clinical deterioration and no follow-up imaging was performed (Table 2). The most common cause of treatment discontinuation across the trametinib/HCQ subgroup was tumor progression (11/19 patients).

**Table 2:** Number of patients evaluable for response, duration of treatment and survival endpoints

		Trametinib/HCQ (*n* = 19)	Trametinib/ Palbociclib (*n* = 15)
Evaluable for response		13	9
	PR	0	1
	PD	13	8
**Not evaluable for response: reason for treatment discontinuation**		**6**	**6**
	Death before staging	1	1
	Patient request	2	0
	Clinical deterioration	3	0
	Toxicity	0	3
	Lost to follow-up	0	2
**Evaluable for duration of treatment**		16	13
	Not evaluable	3	2
**Evaluable for PFS**		17	11
	Not evaluable	2	4
**Evaluable for OS**		15	6
	Not evaluable	4	9

Toxicity was reported for 11 out of 19 patients in the THCQ cohort. In 4 out of the 19 patients, toxicity of CTCAE grade 3 was reported, with nausea grade 3 reported in two patients, and diarrhea, exanthema, and hemolytic uremic syndrome (HUS) grade 3 reported in one patient each. For 8 out of 19 patients no data on toxicity was available.

### Treatment and outcome in the trametinib/palbociclib (TP) cohort

15 out of 34 patients were treated with TP. The most common doses of trametinib and palbociclib in this subgroup were 1 mg/d and 75 mg/d, respectively, with 10 out of 15 patients being administered these doses throughout their treatment; 4 out of 15 patients received trametinib and palbociclib at 2 mg/d and 125 mg/d throughout. In 6 out of 15 patients, the treatment with TP was interrupted, while another 6 out of 15 patients received TP without interruption (3/15 patients: n.a.).

The median duration of treatment in the TP subgroup was 60 days, with the longest duration of TP treatment being 160 days (range 6-160 days; 2/15 patients: n.a.). The median PFS in the TP subgroup was 56 days (range 6-119 days; 95% CI: 47.7–64.3; 4/15 patients: n.a.) (Fig. 1C). Overall survival data was available for only 6 out of 15 patients, with the median OS being 195 days (range 6-453 days; 95% CI: 149.7–240.3; 9/15 patients: n.a.) (Fig. 1D). 9 out of 15 patients were evaluable for response. For one of these patients, a partial remission (PR) was reported as best response, while another patient achieved a clinical benefit despite radiographic progression. For 8 out of 15 patients, progression was reported as best response, while for 6 out of 15 patients, no response data was available. Of the six patients where no response data was available, one had died just six days after initiation of treatment with TP; in three patients, treatment was discontinued due to toxicity without follow-up staging; and two patients were lost to follow-up (Table 2).

Toxicity was reported in 9 out of 15 patients, with toxicity of CTCAE grades 3 and 4 occurring in two patients: one patient with acute pancreatitis grade 4 and rash grade 3, and another patient with thrombocytopenia grade 3. For 6 out of 15 patients, no data on toxicity was available.

In the TP subgroup, two patients derived benefit from treatment and are therefore discussed in more detail below.

#### Case 1

A 53-year-old female, who received prior treatment with FOLFIRINOX received trametinib at 2 mg/d and palbociclib at 125 mg/d in the second-line setting. Molecular analysis had identified a KRAS-G12D mutation, a deletary CDKN2A mutation (p.R58*) as well as mutations in TP53 and ARID1A. After five weeks of treatment, the tumor marker Ca19-9 showed a 90% reduction. Furthermore, the patients eye vision improved as a known choroideal metastasis decreased in size. However, the patient suffered three episodes of recurrent acute pancreatitis during treatment with TP, the most severe being graded as CTCAE grade 4 and the doses of trametinib and palbociclib were reduced to 1 mg/d and 75 mg/d respectively. After three months of treatment, the patient showed radiographic disease progression and rising tumor marker levels, so treatment with TP was discontinued. The total treatment duration with TP was 98 days. She went on to receive gemcitabine/nab-paclitaxel as third-line treatment.

#### Case 2

A 60-year-old female had received two prior lines of treatment before TP and was treated with trametinib at 1 mg/d and palbociclib at 75 mg/d. Molecular analysis had identified a KRAS-G12V mutation, a deletary CDKN2A mutation (p.R58*) as well as a mutation in TP53. After seven weeks of treatment, staging revealed disease progression with appearance of new liver metastases; however, the patient reported improvement of clinical symptoms and thus treatment with TP was continued for a total treatment duration of 160 days until further radiological progression. She went on to receive treatment with oxaliplatin/5-FU/folinic acid (OFF) followed by a rechallenge with nal-irinotecan/5-FU and died eleven months after initiation of trametinib/palbociclib.

## Discussion

To our knowledge, this case series presents the most comprehensive real-world experience of MEK inhibition in combination with either hydroxychloroquine or a CDK4/6 inhibitor in patients with advanced PDAC. The patients in this case series were treated with THCQ or TP based on molecular-informed treatment recommendations. These recommendations were based either on the presence of KRAS mutations (THCQ) or KRAS mutations with concomitant loss of CDKN2A/B (TP). We identified as much as 167 MTB recommendations in Germany for either THCQ or TP across eight cancer centers, highlighting the need for new treatment options for late-stage PDAC; however, most of these recommendations did not result in an actual treatment of patients. As outlined above and discussed below, the results in our cohort do not support the use of either THCQ or TP in patients with advanced pancreatic cancer.

Our results contrast with the previously published data, especially with case reports regarding tumor remissions of heavily pretreated PDAC patients under treatment with THCQ (Kinsey et al. 2019; Xavier et al. 2021; Wu et al. 2021), as well as with data by Rahib et al. reporting on 9 additional evaluable patients with metastatic PDAC treated with HCQ in combination with trametinib or cobimetinib out of which 5 patients achieved stable disease for at least 8 weeks (Rahib et al. 2020).

Previous data on the use of HCQ in pancreatic cancer had been less positive. A phase II trial by Wolpin et al. showed no efficacy of treatment with HCQ monotherapy in patients with previously treated pancreatic cancer (Wolpin et al. 2014). Several trials have investigated different combinations of HCQ with chemotherapy in different stages of PDAC. A phase II trial by Karasic et al. prospectively evaluated gemcitabine/nab-paclitaxel with or without HCQ in a cohort of 112 patients with metastatic PDAC; this study did not improve its primary end point, OS at 12 months, but reported an improved overall response rate (ORR) of 38.2% in the HCQ + chemotherapy group versus 21.1% for chemotherapy alone, leading to the hypothesis that HCQ might improve outcomes in the pre-operative treatment of resectable PDAC (Karasic et al. 2019). This approach was evaluated by Zeh et al. in a single-center prospective trial, randomizing 64 patients with resectable PDAC towards 2 cycles of gemcitabine/nab-paclitaxel with or without HCQ, followed by resection. This study reached its primary endpoint, histopathologic response according to Evans grade, with a significant improvement in the HCQ + chemotherapy group (*p* = .00016), however PFS and OS did not differ between the groups (Zeh et al. 2020).

Regarding combined treatment with HCQ and MEK inhibition, it is worth mentioning recent data by Mehdi et al. pointing towards better efficacy of this approach in PDAC patients with a KRAS-G12R mutation. In this trial, 10 patients with KRAS-mutated PDAC, out of which 6 had a KRAS-G12R mutation, were treated with trametinib or cobimetinib in combination with HCQ; out of 8 patients with evaluable response, one patient with KRAS-G12R showed a partial response and three patients with KRAS-G12R achieved stable disease (Mehdi et al. 2022). However, 5 out of 10 patients in this study had thus far received only one previous line of treatment, so these results may not be representative of heavily pretreated cohorts of patients. KRAS-G12R has some distinct signaling properties than other KRAS isoforms, not interacting with PI3Kα and thus showing enhanced susceptibility towards MEK-ERK inhibition, as well as showing impaired activation of macropinocytosis, which has been described as a resistance mechanism towards autophagy inhibition (Hobbs et al. 2020). Of note, a phase II trial of the MEK inhibitor selumetinib in pretreated patients with KRAS-G12R mutated PDAC showed disease stabilization for more than six months in 3 out of 8 patients (Kenney et al. 2021). In another phase II trial, 6 patients with heavily pretreated KRAS-G12R mutated PDAC all showed a clinical benefit under treatment with cobimetinib in combination with gemcitabine, with 1/6 patients achieving a partial response and 5/6 patients showing disease stabilization, with a median PFS of 6 months in this cohort (Ardalan et al. 2021). However, more clinical data is required to define the impact of KRAS-G12R on the treatment of PDAC, and to establish a possible role of MEK and autophagy inhibition in this subset of PDAC patients. Our own cohort included only one patient with a KRAS-G12R mutation receiving treatment with THCQ, who did not respond to treatment and died 35 days after initiation of THCQ.

The inefficacy of both trametinib/HCQ and trametinib/palbociclib in our case series is in line with another recently published retrospective analysis by Tang et al., who reported on a cohort of 13 PDAC patients with at least two previous lines of treatment receiving trametinib in combination with either HCQ or a CDK4/6 inhibitor (palbociclib or abemaciclib); only one patient in this cohort achieved disease stabilization during treatment with trametinib/HCQ, and in 5 out of 10 evaluable patients toxicities of CTCAE grade 3 or 4 were reported (Tang et al. 2023). Our own toxicity results may even underestimate the toxicity of these treatment regimens due to lack of proper documentation and underreporting of toxicity inherent to our multicentric real-world approach of data generation.

Several phase I and phase II clinical trials are currently underway to further investigate these or similar combination treatments in PDAC patients. The phase I THREAD trial (NCT03825289) as well as the phase II PaTcH trial (NCT05518110) are both evaluating trametinib in combination with HCQ in pretreated advanced pancreatic cancer patients. Another phase I trial is currently investigating the use of the MEK inhibitor binimetinib in combination with HCQ in KRAS-mutated pancreatic cancer patients with at least one prior treatment line for metastatic disease (NCT04132505). Regarding CDK4/6- and MEK-ERK-targeted treatment regimens, a phase I/II trial is currently investigating the ERK inhibitor ulixertinib in combination with palbociclib in different tumor entities, including metastatic pancreatic cancer and melanoma based on promising preclinical data (Goodwin et al. 2023); however, this trial does not require any specific mutational profile in the pancreatic cancer expansion cohort (NCT03454035). The combination of binimetinib and palbociclib is currently evaluated by the phase II ComboMATCH trial across several tumor entities, including an advanced pancreatic cancer cohort (NCT05554367).

Taken together, our results strongly imply that these experimental regimens may not be as effective as initially thought. At this point, it remains speculative as to whether subgroups of metastatic PDAC patients exist that derive benefit from the treatment regimens covered here. Our data argue against the further use of HCQ in combination with MEK or CDK4/6 inhibitors outside of clinical trials. Furthermore, our work highlights the importance of structured and collaborative outcomes research in the setting of Molecular Tumor Boards and precision oncology programs to ensure the reporting of ineffective or potentially harmful therapeutic interventions.

## Data Availability

The data that support the findings of this study are not openly available due to reasons of sensitivity and are available from the corresponding author upon reasonable request.
